# First-line management of necrotizing herpetic retinitis by prioritizing the investigation of immune status and prognostic factors for poor visual outcomes

**DOI:** 10.1007/s10792-023-02656-8

**Published:** 2023-03-15

**Authors:** Emmanuelle Loubsens, Raphaël Adam, Alexa Debard, Lisa Barioulet, Fanny Varenne, Pierre Fournié, Thomas Sales de Gauzy, Priscille Ollé, Guillaume Martin-Blondel, Vincent Soler

**Affiliations:** 1 Ophthalmology Department, Pierre-Paul Riquet Hospital, Toulouse University Hospital, CHU Toulouse, Place Baylac, 31059 Toulouse, France; 2 Department of Infectious and Tropical Diseases, Toulouse University Hospital, Toulouse, France; 3 University of Toulouse III, Toulouse, France; 4 INSERM U1043-CNRS UMR 5282, Centre for Physiopathology of Toulouse-Purpan, Toulouse, France

**Keywords:** Necrotizing herpetic retinitis, Immune status, Antiviral therapy, Retinal complications

## Abstract

**Purpose:**

To review management, treatment, and outcomes of patients with necrotizing herpetic retinitis (NHR) to propose an algorithm for first-line management of NHR.

**Methods:**

Retrospective evaluation of a series of patients with NHR at our tertiary center between 2012 and 2021 using demographic, clinical, ophthalmologic, virological, therapeutic, and prognostic characteristics was performed. Patients were classified by NHR type: acute retinal necrosis (ARN), progressive outer retinal necrosis (PORN), cytomegalovirus (CMV) retinitis.

**Results:**

Forty-one patients with NHR were included: 59% with ARN, 7% with PORN, and 34% with CMV retinitis. All patients with CMV retinitis and PORN were immunocompromised versus 21% of patients with ARN. CMV infection was found in 14 (34%) patients, varicella zoster virus infection in 14 (34%) patients, herpes simplex virus type 2 infection in 8 (20%) and type 1 infection in 5 (12%) patients. Intravenous antiviral therapy was received by 98% of patients and intravitreal antiviral injections by 90% of patients. The overall complication rate during follow-up was 83% of eyes. Most frequent complications were retinal detachment (33% eyes) and retinal break (29% eyes). Prognostic factors for poor visual outcomes were pre-existing monocular vision loss in contralateral eye among 17% of patients, bilateral NHR in 17% of patients, posterior pole involvement in 46% of eyes, and involvement > 2 retinal quadrants in 46% of eyes.

**Conclusions:**

The visual prognosis of patients with NHR remains poor. Prompt investigation of immune status and presence of factors justifying intravitreal antiviral injections must be prioritized to initiate and adapt management while awaiting causative virus confirmation.

**Supplementary Information:**

The online version contains supplementary material available at 10.1007/s10792-023-02656-8.

## Introduction

Necrotizing herpetic retinitis (NHR) refers to infection by viruses of the *Herpesviridae* family and is induced by herpes simplex virus type 1/2 (HSV1/2), varicella zoster virus (VZV), cytomegalovirus (CMV), and more rarely by Epstein Barr virus (EBV) [1]. NHR comprises three different and severe disease entities distinguished by their clinical course and distinctive patterns of retinal opacification: (1) Acute retinal necrosis (ARN) is found primarily in immunocompetent patients [2, 3]. The classic triad of ARN [4, 5] associates the sudden onset of peripheral retinal necrosis with centripetal and circumferential progression, predominant arterial occlusive vasculitis, and inflammatory involvement of the anterior and posterior segments (Supplementary Fig. 1). (2) Progressive outer retinal necrosis (PORN) is characterized by a rapid progression of external retinal necrosis with multifocal, transmural, and centrifugal evolution [6]. PORN does not frequently present with retinal hemorrhage or vasculitis, but is predominantly bilateral and found in immunocompromised patients (Supplementary Fig. 2) [7]. (3) CMV retinitis is characterized by the combination of fluffy white patches of retinal necrosis that spread centrifugally with retinal hemorrhage and vascular engorgement (Supplementary Fig. 3). Development is slower than ARN or PORN, and CMV retinitis predominantly affects HIV-infected patients, but can be found in other forms of immunodeficiency [8, 9].

PCR-based assays on ocular samples can frequently identify the causative viral infection [8, 10], but the visual prognosis of patients with NHR remains poor despite the broad availability of antiviral therapy for herpes treatment [11]. Thus, the positive diagnosis of NHR and the requirement for prompt treatment initiation to reduce visual loss is often based on clinical suspicion. Moreover, NHR remains rare, resulting in a lack of both large-scale studies and standard treatment guidelines [12].

Here, we report on our management of NHR in our tertiary ophthalmology center. We performed a retrospective evaluation on a series of patients with NHR over a ten-year period using demographic, clinical, ophthalmologic, virological, therapeutic, and prognostic characteristics with the goal of putting forward an algorithm for first-line management options of NHR.

## Materials and methods

### Study design

We retrospectively reviewed all patients referred to our university hospital ophthalmology department with chorioretinitis, chorioretinal diseases in other infectious diseases (code H320 in the French PMSI database), and herpetic eye diseases from January 1, 2012 to December 31, 2021. An official waiver of ethical approval was granted from the IRB of Toulouse University Hospital given the retrospective nature of the study as asserted by French Jardé law (study reference: RnIPH 2021–110). All procedures performed were part of routine care, in accordance with institutional guidelines and with the principles and regulations of the Declaration of Helsinki. Patients or their legal guardians/relatives received clear written information and gave free and written informed consent to participate. All data have been anonymized for publication purposes.

### Patient selection and data collection

Patients with inflammatory diseases of the posterior segment (foci, optic disk edema, vasculitis) testing positive for viral retinitis after anterior chamber paracentesis were included for study. The baseline data concerning patient demographic and clinical characteristics, ophthalmologic characteristics and outcomes, and antiviral treatments were collected. Clinical characteristics included history of herpes infection, onset of NHR, delay between NHR onset and consultation/diagnosis, presence of immunodeficiency and type, and follow-up time. Ophthalmologic characteristics and outcomes included initial and final visual acuity using the logarithm of the minimum angle of resolution (logMAR) scale, fundus features (retinography capturing the posterior pole/periphery were available for all patients included), complications, surgical interventions, and recurrences. Para-clinical data analyzed included retinal angiography, OCT evaluation, and biological assessments.

### Diagnosis of NHR type

Patients with NHR were classified into ARN, PORN, and CMV retinitis after three independent and blinded interpretations of clinical criteria (by EL, RA, and VS). The diagnosis of ARN was based on criteria from the recent Standardization of Uveitis Nomenclature (SUN) Working Group [13]. Immunocompromised cases of PORN were diagnosed based on rapidly progressive, multifocal, and deep retinal opacifications involving both the peripheral retina and the posterior pole [14]. Immunocompromised cases of CMV retinitis were diagnosed according to recent SUN Working Group criteria for CMV retinitis [15]. We checked for the signs of immune recovery uveitis among HIV-infected patients with CMV retinitis [16].

### Statistical analyses

Qualitative variables are presented by frequency/percentage and quantitative variables by average ± standard deviation. Comparisons between continuous variables from two groups were made using the Mann–Whitney *U* test, and the Kruskal–Wallis test was used for comparisons between more than two groups. Each eye was considered as independent for statistical analyses. The significance threshold retained was the classic 5% threshold (*p* < 0.05). Statistical analyses were performed using STATA 11.2 software (StataCorp, Texas).

## Results

### Demographic characteristics

Out of 435 patients referred to us with suspected inflammatory diseases of the posterior segment, 40 (9%) patients tested positive for viral retinitis via anterior chamber paracentesis. Note that, one negative case was already under antiviral treatment with a history of CMV retinitis and was also included for study; thus leading to inclusion of 41 patients (48 eyes) diagnosed with NHR. Among these, 28 (68%) were of male sex and the sex ratio was 2.2. The average age at diagnosis was 53.2 ± 20 years [4–92 years]. Thirteen (32%) patients had a history of herpes viral infection. This was non-ocular for 4 (31%) patients (lip, *n* = 2; herpes zoster on leg, *n* = 1; herpes meningoecephalitis, *n* = 1) or ocular for 9 (69%) patients: herpetic uveitis (*n* = 1), herpes zoster ophthalmicus (*n* = 1), or a previous episode of NHR (*n* = 7; 2 were under systemic anti-CMV therapy at inclusion). The average delay between first clinical signs and the etiological diagnosis of NHR was 16 ± 7 days [0–62 days]. A delay in diagnosis was defined as > 5 days between onset of symptoms and consultation; this occurred for 27 (69%) patients and related to consultation postponement by the patient among the majority of cases (66%). A delay in antiviral treatment was observed in 9 (22%) patients due to lack of knowledge and/or availability of the diagnosis by the ophthalmologist (absence of pupillary dilatation/poor fundus visibility). This delay was on average 14 ± 12 days [7–62 days].

### Ophthalmologic characteristics

Twenty-four (59%) patients (*n* = 26 eyes; 54%) were diagnosed with ARN, three (7%) patients (*n* = 4 eyes; 8% eyes) with PORN, and 14 (34%) patients (*n* = 18 eyes; 38%) with CMV retinitis. There was no difference in mean age between the patients according to NHR type (ARN: 52 years; PORN: 53 years; CMV retinitis: 55 years (*p* = 0.96)). Table 1 summarizes the ophthalmologic characteristics of our patient series. Seven (17%) patients with NHR (unilateral) had monocular vision with complete or almost complete vision loss in the contralateral eye (*n* = 4 with ARN, *n* = 3 with CMV retinitis). Loss of vision was due to amblyopia (*n* = 1), previous retinal detachment (*n* = 5), or previous ocular trauma (*n* = 1). NHR was bilateral in 7 (17%) patients: *n* = 2 with ARN, *n* = 1 with PORN, and *n* = 4 with CMV retinitis. Bilaterality was initial in five and secondarily in two cases. The average initial visual acuity was 1.04 ± 0.99 logMAR: 1.07 ± 0.94 logMAR for eyes with ARN, 1.53 ± 1.33 logMAR for eyes with PORN, and 0.68 ± 0.65 logMAR for eyes with CMV retinitis. There was no difference in initial visual acuity between the three types of NHR (*p* = 0.39). Slit lamp examination (anterior chamber cells (Tyndall scattering), keratic precipitates, iris-lens synechia) revealed anterior segment inflammation in a total of 42 (88%) eyes: among all eyes (*n* = 26;100%) with ARN, all eyes (*n* = 4; 100%) with PORN, and 12 (67%) eyes with CMV retinitis. Intraocular pressure measured using a Goldmann Applanation Tonometer gave an average of 16.4 mmHg: 16.8 mmHg in eyes with ARN, 12 mmHg with PORN, and 16.7 mmHg with CMV retinitis. Intraocular pressure was high (> 21 mmHg) in seven (15%) eyes, but the character of the hypertension was transient with a total regression after antiviral treatment.

**Table 1:** Patient ophthalmologic characteristics according to type of necrotizing herpetic retinitis or all types combined

NHR type	ARN	PORN	CMV retinitis	Total
**Number of patients**, *n* (% total patients)	**24 (59%)**	**3 (7%)**	**14 (34%)**	**41**
Pre-existing monocular vision, *n* (% by group)	4 (17%)	0	3 (21%)	7 (17%)
Bilateral NHR, *n* (% by group)	2 (8%)	1 (33%)	4 (29%)	7 (17%)
**Number of eyes**, *n* (% total eyes)	**26 (54%)**	**4 (8%)**	**18 (38%)**	**48**
Average initial visual acuity (logMAR)	1.07 ± 0.94	1.53 ± 1.33	0.68 ± 0.65	1.04 ± 0.99
Anterior segment inflammation, *n* (% by group)	26 (100%)	4 (100%)	12 (67%)	42 (88%)
Anterior chamber cells (Tyndall Scattering), *n* (% by group) 1 + 2 + 3 + 4 +	5 (19%) 7 (27%) 6 (23%) 2 (7%)	1 (25%) 1 (25%) 1 (25%) 0	3 (17%) 3 (17%) 3 (17%) 0	9 (19%) 11 (23%) 10 (21%) 2 (4%)
Keratic precipitates, *n* (% by group)	24 (92%)	4 (100%)	9 (50%)	37 (77%)
Iris-lens synechia, *n* (% by group)	5 (19%)	1 (25%)	2 (11%)	8 (17%)
Average intraocular pressure (mmHg)	16.8	12	16.7	16.4
Vitritis, *n* (% by group)	22 (85%)	4 (100%)	14 (78%)	40 (83%)
Vasculitis, *n* (% by group)	16 (62%)	4 (100%)	10 (56%)	30 (63%)
Optic disk edema, *n* (% by group)	9 (35%)	1 (25%)	3 (17%)	13 (27%)
Foci of retinal necrosis, *n* (% by group)	22 (85%)	4 (100%)	15 (83%)	41 (85%)
Retinal hemorrhage, *n* (% by group)	17 (65%)	4 (100%)	12 (67%)	33 (69%)
Extent of retinitis, *n* (% by group)				
Limited to one quadrant	7 (27%)	0	4 (22%)	11 (23%)
Limited to two quadrants	7 (27%)	1 (25%)	6 (33%)	14 (29%)
> two quadrants	12 (46%)	2 (50%)	8 (44%)	22 (46%)
Posterior pole location, *n* (% by group)	8 (31%)	4 (100%)	10 (56%)	22 (46%)

The use of bold in the main headings of table is to highlight the main characteristics and then groups within via subheadings (not in bold). The values of n (%) in the sub-headings are therefore calculated from values presented in the main headings (patient or eye numbers)

*ARN*: acute retinal necrosis; *CMV*: cytomegalovirus; logMAR: logarithm of the minimum angle of resolution; *NHR*: necrotizing herpetic retinitis; *PORN*: progressive outer retinal necrosis

Regarding the fundus, vitritis was present in a total of 40 (83%) eyes, vasculitis in 30 (63%) eyes, optic disk edema in 13 (27%) eyes, confluent patches of retinal necrosis in 41 (85%) eyes, and retinal hemorrhage in 33 (69%) eyes. The retinitis was limited to one quadrant in a total of 11 (23%) eyes (27% of eyes with ARN, 0% with PORN, and 22% with CMV retinitis), two quadrants in 14 (29%) eyes (27% of eyes with ARN, 25% with PORN, and 33% of eyes with CMV retinitis), and more than two quadrants in 22 (46%) eyes (46% of eyes with ARN, 50% with PORN, and 44% with CMV retinitis). A predominant involvement of the posterior pole was present in 22 (46%) eyes: 8 (31%) eyes with ARN, 4 (100%) with PORN, and 10 (56%) with CMV retinitis. The retinal fundus was not accessible for one eye with ARN due to retinal detachment. All remaining eyes with ARN (52%, *n* = 25) presented with peripheral involvement alone or in addition to involvement of the posterior pole.

### Clinical characteristics

In terms of immune status, five patients diagnosed with ARN (21%) were immunocompromised compared to all patients with CMV retinitis (100%, *n* = 14) and PORN (100%, *n* = 3) (*p* = 0.001). The different forms of immunodeficiency are summarized in Table 2. Four patients had a history of diabetes at initial examination: One with ARN had controlled type 1 diabetes, one with PORN had controlled or uncontrolled (missing data) type 2 diabetes, and two with CMV retinitis had corticosteroid-induced diabetes; one with uncontrolled diabetes induced following treatment of Myasthenia Gravis and one with controlled diabetes following treatment after bone marrow transplantation. Briefly, hematology treatments were mainly for blood cancers (*n* = 6), then kidney transplantation-associated immunosuppressive drugs (*n* = 3) or kidney transplantation-associated hematological disorders (*n* = 2), or for Crohn’s disease (*n* = 1). It is noteworthy that none of the HIV-infected patients with CMV retinitis (*n* = 5) developed immune recovery uveitis during follow-up (despite three achieving immune recovery).

**Table 2:** The different forms of immunodeficiency in our patient series

NHR type	ARN	PORN	CMV retinitis	Total
**Number of patients (*n*)**	**24**	**3**	**14**	**41**
**Immunocompromised patients, *n* (% by group)**	**5 (21%)**	**3 (100%)**	**14 (100%)**	**22 (54%)**
Forms of immunodeficiency, *n* (% by group)				
HIV	0	1 (33%)	5 (36%)	6 (27%)
Systemic corticosteroid therapy	3 (60%)	1 (33%)	3 (21%)	7 (32%)
Immunosuppressive therapy	1 (20%)	1 (33%)	4 (29%)	6 (27%)
Solid cancer chemotherapy	1 (20%)	1 (33%)	4 (29%)	6 (27%)
Diabetes	1 (20%)	1 (33%)	2 (14%)	4 (18%)
Type 1, *n*	1	0	0	
Type 2, *n*	0	1	0	
Secondary diabetes, *n*	0	0	2	
Organ transplantation	2 (40%)	1 (33%)	3 (21%)	6 (27%)
Hematology treatment	3 (60%)	1 (33%)	8 (57%)	12 (55%)
Multifactorial immunodeficiency	3 (60%)	3 (100%)	8 (57%)	14 (64%)

The use of bold in the main headings of table is to highlight the main characteristics and then groups within via subheadings (not in bold). The values of n (%) in the sub-headings are therefore calculated from values presented in the main headings (patient or eye numbers)

*ARN*: acute retinal necrosis; *CMV*: cytomegalovirus; *HIV*: human immunodeficiency virus; *NHR*: necrotizing herpetic retinitis; *PORN*: progressive outer retinal necrosis

Virological diagnosis was carried out by anterior chamber puncture in all patients. PCR results were positive for 40/41 cases (the negative case was already under antiviral treatment with a history of CMV retinitis). Fourteen (34%) patients tested positive for VZV infection, 5 (12%) for HSV1 infection, 8 (20%) for HSV2 infection, and 14 (34%) for CMV infection. Supplementary Fig. 4 summarizes the different viral infections according to NHR type. A patient with unilateral ARN demonstrated the presence of papillitis, vasculitis, and choroidal foci (Supplementary Fig. 5). We also had a case of Kyrieleis arteritis in a patient with VZV ARN (Supplementary Fig. 6).

### Treatments

All patients except one (98%) were treated by intravenous (IV) antiviral therapy: 20 (50%) patients received aciclovir alone (10 mg/kg/8 h), 8 (20%) ganciclovir alone (2.5 mg/kg/12 h), and 3 (8%) foscarnet alone (90 mg/kg/12H). One patient with CMV retinitis initially under IV ganciclovir switched to injections of cidofovir due to resistance. Nine (22%) patients received dual therapy: Two received aciclovir and foscarnet in attack treatment, six received aciclovir and ganciclovir, and one ganciclovir and foscarnet. The average IV treatment duration was 16.9 ± 5.5 days [7–30 days]. One patient was contraindicated IV therapy due to a mental disorder and so received oral valaciclovir in first line. Table 3 summarizes the different antiviral treatments undertaken.

**Table 3:** Antiviral treatments undertaken by patients according to the different types of necrotizing herpetic retinitis or all types combined

NHR type	ARN	PORN	CMV retinitis	Total
**Number of patients (n)**	**24**	**3**	**14**	**41**
**IV therapy, *n* (% by group)**	**23 (96%)**	**3 (100%)**	**14 (100%)**	**40 (98%)**
Aciclovir	20 (87%)	0	0	20 (50%)
Ganciclovir	0	0	8 (57%)	8 (20%)
Foscarnet	0	0	3 (21%)	3 (8%)
Aciclovir + foscarnet	0	1 (33%)	1 (7%)	2 (5%)
Aciclovir + ganciclovir	2 (9%)	3 (100%)	1 (7%)	6 (15%)
Ganciclovir + foscarnet	0	0	1 (7%)	1 (3%)
**Relay with oral antiviral therapy, *n* (% by group)**	**23 (96%)**	**3 (100%)**	**13 (93%)**	**39 (95%)**
Aciclovir	1 (4%)	0	0	1 (3%)
Valaciclovir	22 (96%)	3 (100%)	0	25 (64%)
Valganciclovir	0	0	13 (100%)	13 (33%)
**Intravitreal injection, *n* (% by group)**	**22 (92%)**	**2 (66%)**	**13 (93%)**	**37 (90%)**
Ganciclovir	17 (77%)	1 (50%)	8 (62%)	26 (70%)
Foscarnet	5 (23%)	1 (50%)	5 (38%)	11 (30%)
**Corticosteroids,** ***n*** **(% by group)**	**24 (100%)**	**2 (66%)**	**2 (14%)**	**28 (68%)**
**Aspirin,** ***n*** **(% by group)**	**19 (79%)**	**0**	**0**	**19 (46%)**

The use of bold in the main headings of table is to highlight the main characteristics and then groups within via subheadings (not in bold). The values of n (%) in the sub-headings are therefore calculated from values presented in the main headings (patient or eye numbers)

ARN: acute retinal necrosis; CMV: cytomegalovirus; IV: intravenous; NHR: necrotizing herpetic retinitis; PORN: progressive outer retinal necrosis

IV treatment was associated with an oral relay with aciclovir or valaciclovir for HSV/VZV infection, or valganciclovir for CMV infection (the patient under injections of cidofovir continued this treatment alone). The average treatment duration was 830 days (from 45 days to for life for 15 patients). Oral treatment dosages were (1) 1 mg/kg aciclovir for one patient (2) 3 g/24 h valaciclovir for 25 patients, (3) twice daily 450 mg valganciclovir for 13 patients, with a gradual decrease over several weeks. Systemic corticosteroids were administered to 28 (68%) patients once control of the infection was established: 17 cases (61%) via flash IV (250–500 mg daily for three days) and 11 (39%) cases were only administered oral treatment at a dose of 1 mg/kg/day. The indications for systemic corticosteroid administration were 10 patients with severe vitritis and 18 patients with papillitis. Supplementary Table 1 summarizes the demographic, clinical and ophthalmologic characteristics, as well as initial intravenous antiviral treatments taken for each patient included.

Thirty-seven (90%) patients additionally received intravitreal injections: 11 (30%) patients received foscarnet and 26 (70%) received ganciclovir. One patient with CMV retinitis initially received foscarnet and then switched to ganciclovir, and one ARN VZV-positive patient switched from ganciclovir to foscarnet. Patients received an average of 4.3 ± 2.1 intravitreal injections [1–9 injections].

### Prognosis and complications

The average follow-up time was 12.8 ± 5.6 months. The average final visual acuity of all eyes included was 0.93 ± 0.75 logMAR: 0.89 ± 0.73 logMAR for eyes with ARN, 1.65 ± 1.34 logMAR for eyes with PORN, and 0.65 ± 0.59 logMAR for eyes with CMV retinitis. There was no significant difference in final visual acuity between NHR type (*p* = 0.34). The overall complication rate was 40 (83%) eyes during follow-up. The two main complications were retinal break (14 eyes, 29%) and retinal detachment (16 eyes, 33%). Retinal break occurred among 9 (35%) eyes with ARN, 1 (25%) with PORN, and 4 (22%) with CMV retinitis. Retinal detachment occurred among 11 (42%) eyes with ARN, 2 (50%) with PORN, and 3 (17%) with CMV retinitis. Four (10%) patients presented with retinal detachment at initial examination and 12 (29%) patients developed secondary retinal detachment (Supplementary Fig. 7). Among the aforementioned 12 patients, 10 (83%) had received intravitreal injections before the onset of retinal detachment. The delay between onset of retinal detachment and diagnosis was 156 ± 138 days. No bilateral retinal detachment was reported. It is noteworthy that retinal detachment occurred in 17% of patients with CMV retinitis, in 40% with VZV retinitis, in 40% with HSV1 retinitis, and in 88% with HSV2 retinitis (data not shown). Macular edema developed in 11 (23%) eyes: among 4 (15%) eyes with ARN, 3 (75%) with PORN, and 4 (22%) with CMV retinitis. Macular epiretinal membrane developed in 12 (25%) eyes: among 9 (35%) eyes with ARN, 1 (25%) with PORN, and 2 (11%) with CMV retinitis. A total of five (10%) eyes developed optic nerve atrophy and nine (19%) secondary vitreous opacities. Regarding unilateral recurrence of NHR, 9 (22%) patients were affected after an average of 807 ± 773 days; *n* = 7/9 (78%) of these patients were under oral antiviral therapy during relapse, all 9 (100%) patients had received intravitreal antiviral injections, and *n* = 4/9 (44%) patients had postponed consultation resulting in a delay in treatment initiation. Table 4 summarizes the prognosis and complications of our patient series.

**Table 4:** Prognosis and complications of included eyes according to the different types of necrotizing herpetic retinitis or all types combined

NHR type	ARN	PORN	CMV retinitis	Total
**Number of eyes (*n*)**	**26**	**4**	**18**	**48**
Average final visual acuity (logMAR)	0.89 ± 0.73	1.65 ± 1.34	0.65 ± 0.59	0.93 ± 0.75
Retinal break, *n* (% by group)	9 (35%)	1 (25%)	4 (22%)	14 (29%)
Retinal detachment, *n* (% by group)	11 (42%)	2 (50%)	3 (17%)	16 (33%)
Macular involvement, *n* (% group)	15 (58%)	4 (100%)	10 (56%)	29 (60%)
Macular edema, *n* (% group)	4 (15%)	3 (75%)	4 (22%)	11 (23%)
Epiretinal membrane, *n* (% group)	9 (35%)	1 (25%)	2 (11%)	12 (25%)
Macular atrophy, *n* (% group)	2 (8%)	0	4 (22%)	6 (13%)
Optic nerve atrophy, *n* (% by group)	2 (8%)	0	3 (17%)	5 (10%)
Vitreous opacities, *n* (% by group)	4 (15%)	1 (25%)	4 (22%)	9 (19%)

The use of bold in the main headings of table is to highlight the main characteristics and then groups within via subheadings (not in bold). The values of n (%) in the sub-headings are therefore calculated from values presented in the main headings (patient or eye numbers)

*ARN*: acute retinal necrosis; *CMV*: cytomegalovirus; logMAR: logarithm of the minimum angle of resolution; *NHR*: necrotizing herpetic retinitis; *PORN*: progressive outer retinal necrosis

Prophylactic treatment of retinal detachment by laser coagulation behind zones of retinal necrosis was performed in 11 (23%) eyes from 11 patients (27%) (all receiving IV therapy and intravitreal injections). Retinal detachment occurred in 4 (36%) of these aforementioned patients (3 eyes with ARN and 1 eye with CMV retinitis).

Finally, 13 patients were operated on for retinal detachment with an average of 2.2 ± 1.2 interventions per patient [1–5 interventions]. Twelve operated patients (92%) underwent vitrectomy with silicone oil tamponade. Silicone oil was removed in 10 (83%) of these patients. Only one patient received gas tamponade. At follow-up end, 8 operated patients (62%) had reapplied retinas (*n* = 6/8 had received intravitreal injections), and 5 (38%) did not have reapplied retinas (*n* = 3/5 (60%) had received intravitreal injections). Final visual acuity was significantly worse for eyes with retinal detachment (1.18 ± 0.97 logMAR) versus eyes without retinal detachment (0.51 ± 0.42 logMAR) (*p* = 0.03); regardless of whether eyes were operated on or not and whether retinas were reapplied or not. Final average visual acuity was bad for operated patients (1.19 ± 1.02 logMAR).

## Discussion

NHR comprises three different but frequently blinding disease entities known as ARN, PORN, and CMV retinitis. These are characterized by their clinical course and distinctive patterns of retinal opacification [2–8]. Despite both accurate PCR-based assays that can identify the causative viral infection [10] and the broad availability of antiviral therapy for herpes treatment, the visual prognosis of patients with NHR remains poor [11] and majorly affected by delay in therapeutic management [17]. Urgent treatment is thus generally introduced based on clinical presumption in order to reduce visual loss. Here, we suggest prioritizing both investigation of immune status and the presence of prognostic factors for poor visual outcomes to adapt initial antiviral treatment while awaiting identification of the causative virus.

In line with previous reports, our series of patients with NHR (*n* = 41) showed a male predominance (sex ratio 2.2) with an onset at approximately 50 years old [2, 18]. We grouped our patients according to diagnosis of NHR type: 24 (59%) patients were diagnosed with ARN, 3 (7%) with PORN, and 14 (34%) with CMV retinitis. Supplementary Fig. 4 indicates the different viral infections according to NHR type in our study: In the PORN group, 75% (*n* = 3) eyes were VZV-infected, and 25% (*n* = 1) eyes were HSV2-infected. In the ARN group, 46% (*n* = 12) eyes were VZV-infected, 23% (*n* = 6) were HSV1-infected, and 31% (*n* = 8) were HSV2-infected. We thus observed the previously reported trend for a predominance of VZV infection among patients with ARN and PORN [1, 19]. In agreement with Kempen et al. [20], none of the five HIV-infected patients with active CMV retinitis developed immune recovery uveitis during follow-up, and this was despite immune reconstitution among three of these patients. More aggressive anti-CMV therapy during the active phase of CMV could reduce the development of immune recovery uveitis [21]. Our three aforementioned patients reaching immune recovery did indeed receive intravitreal injections of ganciclovir/foscarnet in addition to IV antiviral therapy. Finally, note that, all eyes with PORN presented with characteristic minimal vitritis at onset [22].

**Fig. 1:**
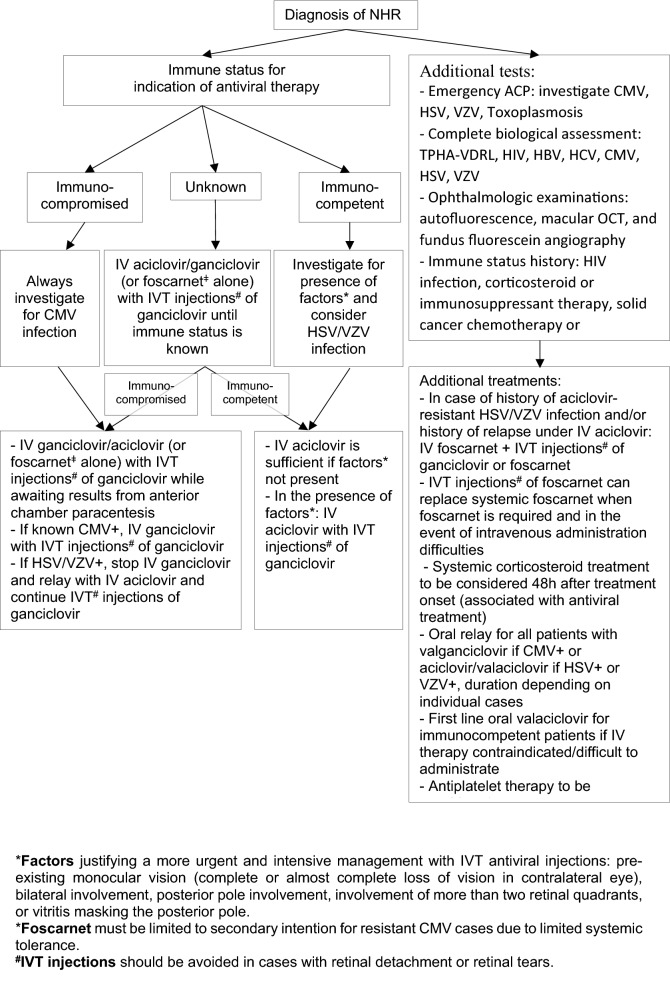
Algorithm for first-line management of patients with necrotizing herpetic retinitis (NHR). Antiviral treatment can be immediately initiated according to patient immune status and the presence or not of factors justifying intravitreal antiviral injections in addition to intravenous antiviral therapy while awaiting results of anterior chamber paracentesis. ACP; anterior chamber paracentesis; CMV: cytomegalovirus; HBV: hepatitis B virus; HCV: hepatitis C virus. HIV; human immunodeficiency virus; HSV: herpes simplex virus; IV: intravenous; IVT: intravitreal; OCT: optical coherence tomography; TPHA-VDRL: Treponema pallidum hemagglutination assay-Veneral Disease Research Laboratory; VZV: varicella zoster virus

Based on the NHR severity factors previously put forward by Tran et al. [18], we identified pre-existing monocular vision (complete or almost complete vison loss in contralateral eye) (17% of patients), bilateral NHR (17% of patients), posterior pole involvement (46% of eyes), and involvement of more than two retinal quadrants (46% of eyes) in our patient series. In terms of the immune status of our patient series, 21% of patients with ARN were immunocompromised versus 100% in both the CMV retinitis and PORN groups. Immunodeficiency and the aforementioned NHR severity factors have already been put forward as prognostic factors for poor visual outcomes requiring more urgent and intensive management with intravitreal antiviral injections in addition to IV antiviral therapy [18]. Therefore, in line with the strategy of not delaying treatment initiation while awaiting identification of the causative virus (in ARN [12]), we suggest for patients with suspected NHR the prompt investigation of (1) immune status and (2) the presence of the aforementioned factors for initiation and adaptation of antiviral therapy while awaiting the results of anterior chamber paracentesis. Hereby, we put forward an algorithm for the first-line management of NHR incorporating current management options (see Fig. 1). Briefly, NHR must be suspected in all patients presenting with uveitis and pupillary dilatation must be performed in order to reduce diagnosis delay. Antiviral treatment can be immediately initiated and adapted while awaiting the results of anterior chamber paracentesis according to patient immune status and the presence of factors justifying more intensive treatment; i.e., intravitreal antiviral injections in addition to IV antiviral therapy for immunocompromised patients or presenting with one of the listed factors. It is noteworthy that we have added vitritis masking the posterior pole as a factor additionally justifying intravitreal antiviral injections. Finally, a large recent study by Takase et al. [23] confirms the association of other factors with poor visual prognosis of patients with ARN: posterior iris synechia, initial visual acuity, VZV infection, and retinal phlebitis. The authors suggest that these factors should be considered for more aggressive therapy. Consideration of these four factors could indeed be beneficial for patients diagnosed with ARN but would not encompass all types of NHR, especially before consideration of the causative virus.

It is noteworthy that consideration of the causative virus during our diagnosis of NHR type according to the SUN criteria for ARN [13] and CMV retinitis [15] resulted in the revision of our original diagnoses of two (5%) patients. We had originally diagnosed one of these patients with PORN and one with ARN based purely on the clinical appearance of the NHR (retinography), but a positive CMV PCR result for both of these patients meant consideration of CMV retinitis. Indeed, in addition to the CMV positivity, both these patients were immunocompromised and presented with indistinct borders of NHR; fulfilling thus the new SUN classification of CMV retinitis [15]. This highlights the potential for misclassification and the differential diagnoses that can be made from virus-confirmed versus virus-unconfirmed criteria for NHR types [13]. On the other hand, this misclassification was only 5% of our patients and was therefore very low. In addition, both patients concerned had received dual IV therapy considering their immunocompromised status (refer to Fig. 1) while awaiting virus confirmation, the CMV infection was thus targeted.

Patients included for study here were treated with classical aciclovir, ganciclovir, and foscarnet antiviral drugs. It is noteworthy that even though IV foscarnet is efficient for the treatment of HSV, CMV, and VZV infections, it is not recommended given its narrow therapeutic index and systemic toxicity, including renal impairment and electrolytic abnormalities [24]. Thus, intravitreal administration of foscarnet must be considered and all the more so when poor prognostic factors are present [24–26]. In our current study, 90% of patients also received intravitreal injections of foscarnet or ganciclovir. Indeed, intravitreal antiviral therapy has become increasingly popular [27, 28] and two comparative studies have already shown beneficial outcomes after combining systemic intravenous and intravitreal antiviral therapy in terms of gain in visual acuity and reduced risk of retinal detachment compared to systemic treatment alone (35% combined versus 60% alone) [26, 29]. To date, foscavir (2.4 mg/0.1 ml) and ganciclovir (2 mg/0.1 ml) are available for intravitreal injection, both administered every three-to-four days until clinical stabilization [27].

The overall complication rate during follow-up was 83% of eyes with NHR in our series. The two main complications were retinal break and retinal detachment. Retinal detachment is developed during follow-up in 33% of patients (83% of these patients had received intravitreal injections). This is indeed lower than rates found in the literature (50–78%) [12], with exception of the Tran et al. [18] study series demonstrating a complication rate of approximately 15%. It is noteworthy that we report a higher rate of retinal detachment in eyes with HSV2 retinitis (88%) (data not shown). Multi-centric studies are required for further investigation of the potential correlation between HSV2 retinitis and retinal detachment. Recently, Risseeuw et al. [30] revealed that 44% of patients with ARN develop retinal detachment despite undergoing prophylactic laser treatment. Likewise, Tibbets et al. [31] demonstrated no difference in the proportion of patients with ARN developing retinal detachment with or without prophylactic laser treatment. In line with these reports, 36% of our patients developed retinal detachment despite undergoing prophylactic laser treatment. The effectiveness of prophylactic laser treatment of retinal detachment remains controversial [32, 33] and must thus be cautiously considered in healthy retina.

Final visual acuity in our patient series showed no difference according to NHR type and was in line or superior to reports in the literature. For instance, in patients with ARN, we found ambulatory final visual acuity in 73% (*n* = 19) eyes versus 36.4% (*n* = 4/11) eyes in the study by Chen et al. [34] (defined by Chen et al. [34] as visual acuity < 1.7 logMAR). Tran et al. [18] found that final visual acuity of patients with ARN and PORN (*n* = 33) was strictly > 1 logMAR in 47% of cases versus 43% of eyes with ARN and PORN (*n* = 30) in our patient series. Finally, Fardeau et al. [2] reported final visual acuity strictly > 1 logMAR for 50–75% of patients versus 27% of patients with ARN in our series. These differences in reports on final visual acuity could be explained by (1) the performance of intensive intravitreal injections, (2) differences in retinal detachment/break rate, (3) differences in initial visual acuity. We cannot rule out that differences could also be partly related to a former lack of a single and clear description of disease type and severity rendering inter-study comparisons difficult. This issue should be solved in the future given the new SUN Working Group classification criteria. Finally, limitations to our study are the retrospective nature, meaning we can also expect some variation in the data collected given the multiple different operators over the 10-year period performing the ophthalmologic examinations.

## Conclusion

The prognosis of patients with NHR remains poor despite antiviral treatments due to retinal complications. Prior diagnosis of both virus or NHR type is not required for our proposed urgent first-line management of NHR, thus reducing delay and improving chances of saving vision.

## Supplementary Information

Below is the link to the electronic supplementary material.

Supplementary file1 (DOCX 28 KB)

Unilateral acute retinal necrosis caused by varicella zoster virus infection in an immunocompetent patient (left-eye retinography): occlusive vasculitis and foci of peripheral retinal necrosis in the inferior and nasal quadrant
Supplementary file2 (TIF 1939 KB)

Bilateral progressive outer retinal necrosis in an immunocompromised patient (retinography)
Supplementary file3 (TIF 4126 KB)

Bilateral cytomegalovirus retinitis in an immunocompromised patient presenting with predominant left retinitis (left-eye retinography)
Supplementary file4 (TIF 1935 KB)

Supplementary file5 (DOCX 46 KB)

Fluorescein (left images) and indocyanine green (right images) angiography in the left eye of a patient presenting with unilateral acute retinal necrosis with papillitis (bottom row), temporal inferior vasculitis (bottom row) and choroidal foci (top row)
Supplementary file6 (TIF 2890 KB)

Kyrieleis arteritis in an immunocompetent patient with unilateral acute retinal necrosis caused by varicella zoster virus infection (right-eye retinography at day 15 of evolution)
Supplementary file7 (TIF 217 KB)

Retinal detachment caused by cytomegalovirus retinitis in the right eye of an immunocompromised patient in our study series (retinography)
Supplementary file8 (TIF 859 KB)
