# Supplementary material for: First-line management of necrotizing herpetic retinitis by prioritizing the investigation of immune status and prognostic factors for poor visual outcomes
Source: Int Ophthalmol. 2023 Mar 15;43(7):2545–56. doi: 10.1007/s10792-023-02656-8 (PMC10313533; doi:10.1007/s10792-023-02656-8)
Supplement: Supplementary file 1 — Supplementary file1 (DOCX 28 KB) [file 10792_2023_2656_MOESM1_ESM.docx]

**Supplementary Table 1**

**The demographic, clinical and ophthalmologic characteristics, and intravenous antiviral treatments taken for the 32 patients with necrotizing herpetic retinitis included for study**

| Patient | Sex | Age range  (years) | Immune status | IVA (OD/OS) logMAR | FVA (OD/OS) logMAR | Virus | Initial IV antiviral treatment | NHR type |
| --- | --- | --- | --- | --- | --- | --- | --- | --- |
| 1 | 1 | Under 10 | + | Impossible^A^ | Impossible^A^ | HSV1 | Aciclovir | ARN |
| 2 | 2 | 10s | + | 0 | 0 | VZV | Aciclovir | ARN |
| 3 | 2 | 10s | + | Impossible^B^ | Impossible^B^ | HSV2 | Oral valaciclovir | ARN |
| 4 | 1 | 20s | + | 0 | 1.3 | HSV2 | Aciclovir | ARN |
| 5 | 1 | 20s | + | 0.2/0.3 | 0.1/2.3 | HSV2 | Aciclovir | ARN |
| 6 | 1 | 30s | + | 0.3 | 0.2 | HSV2 | Aciclovir | ARN |
| 7 | 1 | 30s | + | 1.3/0.2 | 1.3/2 | HSV2 | Aciclovir | ARN |
| 8 | 1 | 30s | + | 0.3 | 0 | VZV | Aciclovir | ARN |
| 9 | 1 | 30s | + | 2 | 0 | HSV1 | Aciclovir | ARN |
| 10 | 1 | 30s | + | 2.3 | 2.3 | VZV | Aciclovir | ARN |
| 11 | 1 | 50s | - | 0.3 | 0.2 | VZV | Aciclovir | ARN |
| 12 | 2 | 50s | + | 2.3 | 1 | HSV1 | Aciclovir | ARN |
| 13* | 1 | 50s | - | 0.3 | 0 | CMV | Aciclovir + foscarnet | CMV retinitis |
| 14 | 1 | 50s | + | 0 | 0 | HSV1 | Aciclovir | ARN |
| 15 | 1 | 50s | + | 2.3 | 1.1 | HSV2 | Aciclovir | ARN |
| 16 | 1 | 60s | + | 0 | 0.1 | VZV | Aciclovir | ARN |
| 17 | 2 | 60s | - | 0.4/0 | 0.2/0 | VZV | Aciclovir | ARN |
| 18 | 2 | 60s | + | 0/1.3 | 0/2.6 | VZV | Aciclovir | ARN |
| 19 | 2 | 70s | + | 2 | 0.2 | VZV | Aciclovir | ARN |
| 20 | 1 | 70s | - | 1/0 | 0.7/0 | VZV | Aciclovir + ganciclovir | ARN |
| 21 | 1 | 70s | + | 0/2.3 | 0.2/2.3 | HSV2 | Aciclovir | ARN |
| 22 | 2 | 80s | + | 2.3 | 2 | VZV | Aciclovir + ganciclovir | ARN |
| 23 | 2 | 80s | - | 0.5 | 1 | VZV | Aciclovir + ganciclovir | ARN |
| 24 | 1 | 80s | - | 2/0 | 2.3/0 | VZV | Aciclovir | ARN |
| 25 | 2 | 90s | + | 1 | 0.3 | HSV1 | Aciclovir | ARN |
| 26 | 1 | 40s | - | 2.3 | 2.6 | HSV2 | Aciclovir + ganciclovir | PORN |
| 27 | 1 | 50s | - | 2.3/2.3 | 2.3/2.3 | VZV | Aciclovir + ganciclovir | PORN |
| 28** | 1 | 50s | - | 1 | 0.3 | CMV | Aciclovir + ganciclovir | CMV retinitis |
| 29 | 1 | 50s | - | 2.3 | 0.7 | VZV | Aciclovir + foscarnet | PORN |
| 30 | 1 | 30s | - | 1 | 0.4 | CMV | Ganciclovir | CMV retinitis |
| 31 | 1 | 30s | - | 2.3/0.3 | 0.7/0 | CMV | Foscarnet | CMV retinitis |
| 32 | 2 | 40s | - | 0.6 | 2.3 | CMV | Ganciclovir | CMV retinitis |
| 33 | 1 | 40s | - | 1.3 | 0 | CMV | Ganciclovir | CMV retinitis |
| 34 | 1 | 40s | - | 0.4/0.7 | 0.4/0.3 | CMV | Ganciclovir | CMV retinitis |
| 35 | 1 | 50s | - | 0/0.4 | 0/0.2 | CMV | Foscarnet | CMV retinitis |
| 36 | 1 | 50s | - | 2.3/0.1 | 2.3/0 | CMV | Ganciclovir | CMV retinitis |
| 37 | 1 | 60s | - | 0/1 | 0/2.3 | CMV | Ganciclovir + foscarnet | CMV retinitis |
| 38 | 2 | 60s | - | 1 | 0.5 | CMV | Foscarnet | CMV retinitis |
| 39 | 2 | 70s | - | 0/0 | 0/0 | CMV | Ganciclovir | CMV retinitis |
| 40 | 2 | 70s | - | 0 | 0.3 | CMV | Ganciclovir | CMV retinitis |
| 41 | 1 | 80s | - | 1 | 2.3 | CMV | Ganciclovir | CMV retinitis |

1: male; 2: female; +: immunocompetent; -: immunocompromised; A: patient too young for measurement; B: patient with a mental disorder contraindicating measurement; ARN: acute retinal necrosis; CMV: cytomegalovirus; FVA: final visual acuity; IV: intravenous; IVA: initial visual acuity; HSV1/2: herpes simplex virus type 1/2; logMAR: logarithm of the minimum angle of resolution; NHR: necrotizing herpetic retinitis; OD: right eye; OS: left eye; PORN: progressive outer retinal necrosis; VZV: varicella zoster virus
