# Supplementary figures and images for: First-line management of necrotizing herpetic retinitis by prioritizing the investigation of immune status and prognostic factors for poor visual outcomes
Source: Int Ophthalmol. 2023 Mar 15;43(7):2545–56. doi: 10.1007/s10792-023-02656-8 (PMC10313533; doi:10.1007/s10792-023-02656-8)

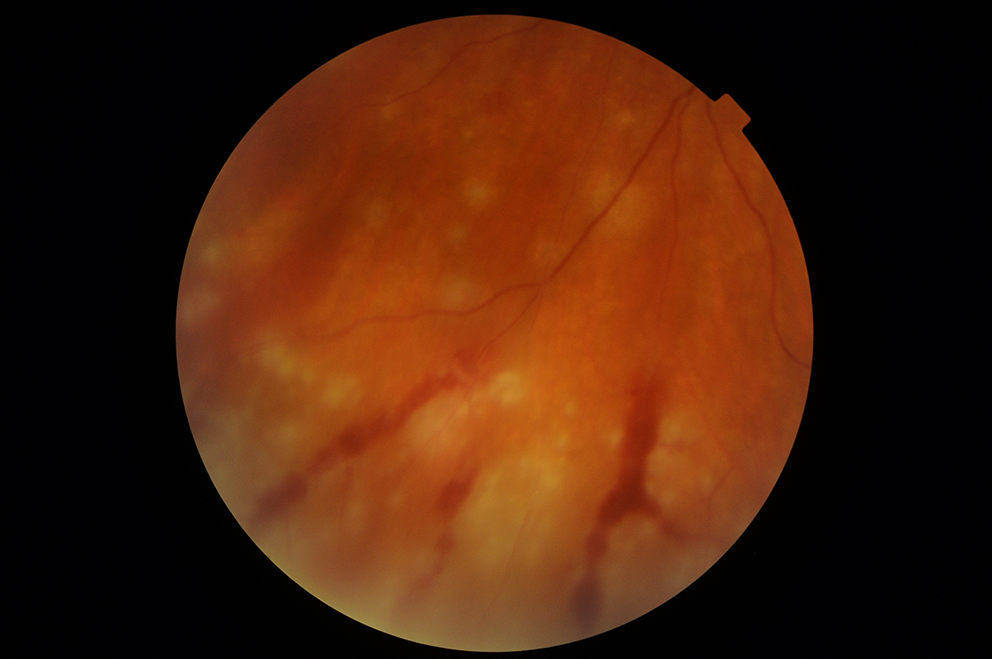

Supplement: Supplementary file 2 — Unilateral acute retinal necrosis caused by varicella zoster virus infection in an immunocompetent patient (left-eye retinography): occlusive vasculitis and foci of peripheral retinal necrosis in the inferior and nasal quadrant Supplementary file2 (TIF 1939 KB) [file 10792_2023_2656_MOESM2_ESM.tif]

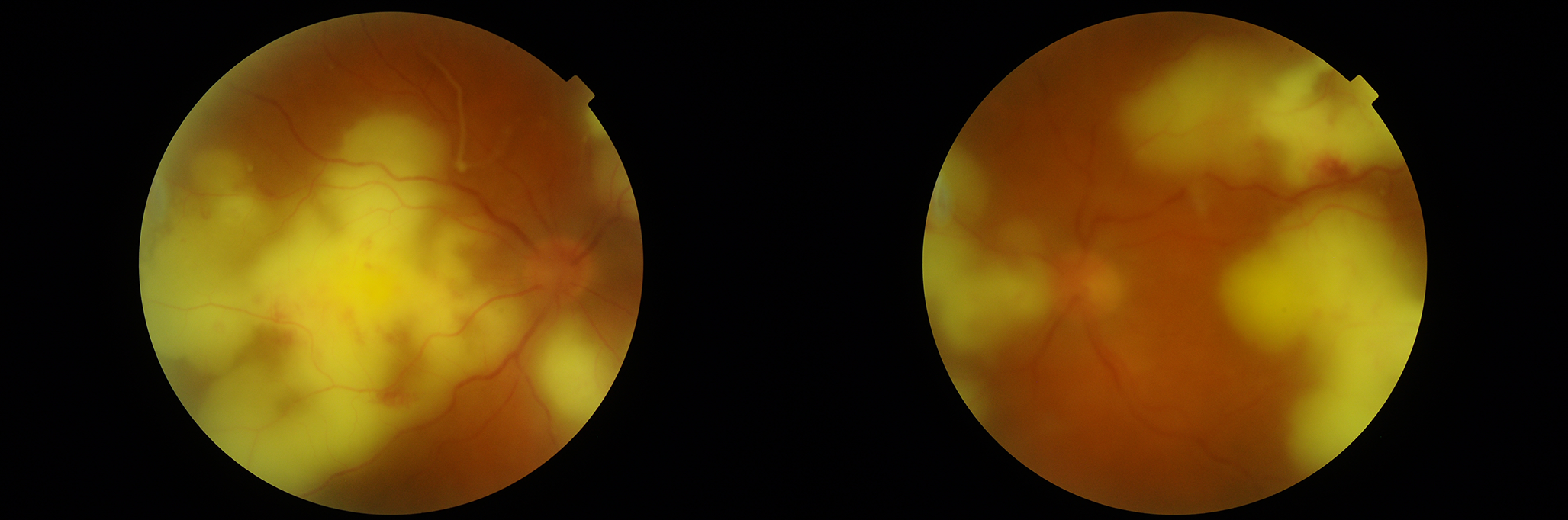

Supplement: Supplementary file 3 — Bilateral progressive outer retinal necrosis in an immunocompromised patient (retinography) Supplementary file3 (TIF 4126 KB) [file 10792_2023_2656_MOESM3_ESM.tif]

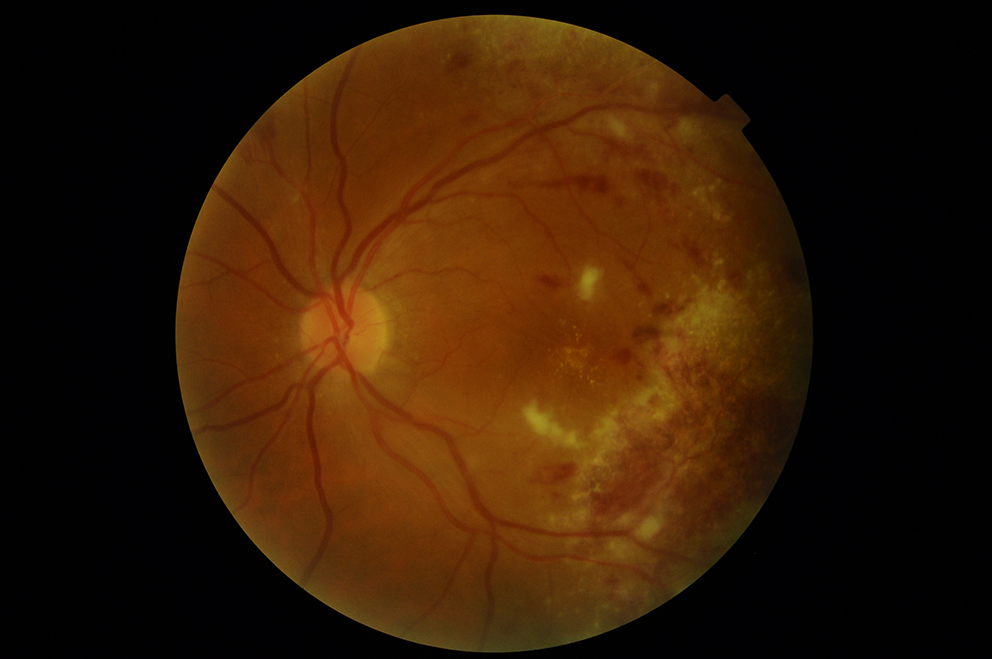

Supplement: Supplementary file 4 — Bilateral cytomegalovirus retinitis in an immunocompromised patient presenting with predominant left retinitis (left-eye retinography) Supplementary file4 (TIF 1935 KB) [file 10792_2023_2656_MOESM4_ESM.tif]

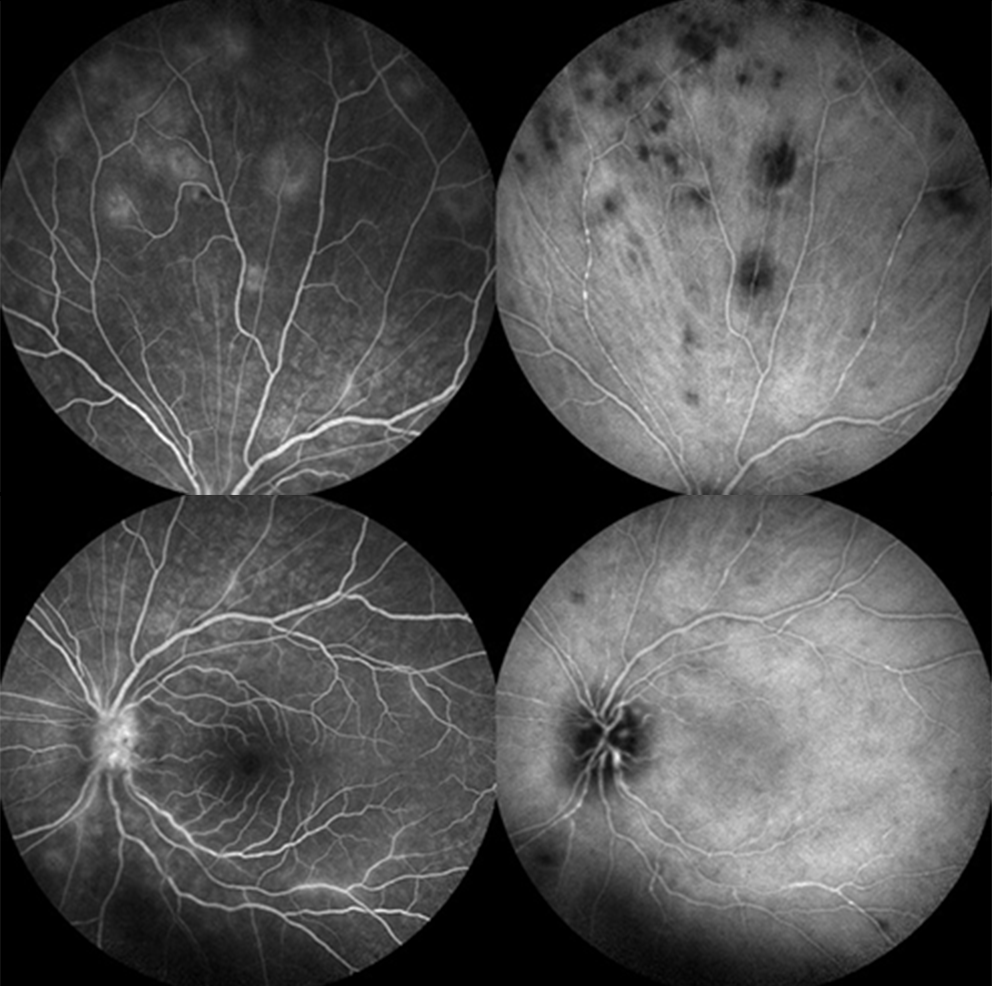

Supplement: Supplementary file 6 — Fluorescein (left images) and indocyanine green (right images) angiography in the left eye of a patient presenting with unilateral acute retinal necrosis with papillitis (bottom row), temporal inferior vasculitis (bottom row) and choroidal foci (top row) Supplementary file6 (TIF 2890 KB) [file 10792_2023_2656_MOESM6_ESM.tif]

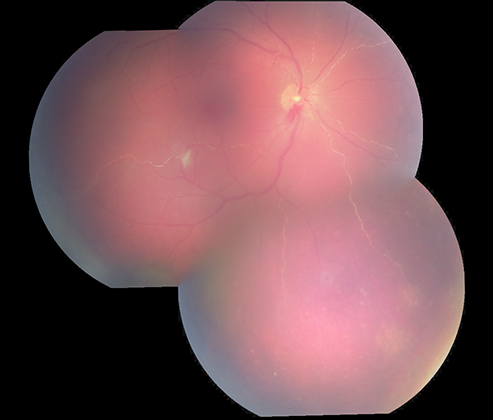

Supplement: Supplementary file 7 — Kyrieleis arteritis in an immunocompetent patient with unilateral acute retinal necrosis caused by varicella zoster virus infection (right-eye retinography at day 15 of evolution) Supplementary file7 (TIF 217 KB) [file 10792_2023_2656_MOESM7_ESM.tif]

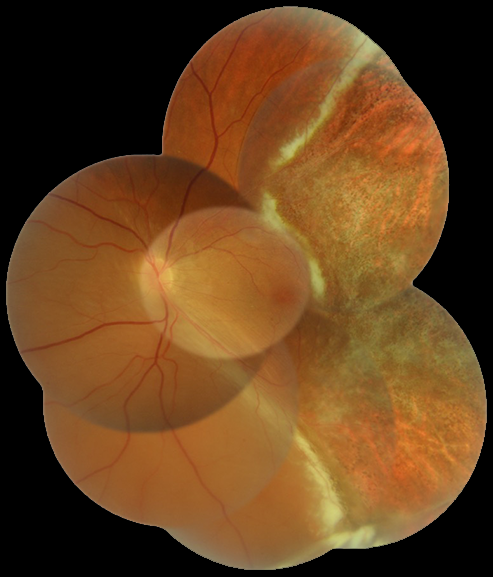

Supplement: Supplementary file 8 — Retinal detachment caused by cytomegalovirus retinitis in the right eye of an immunocompromised patient in our study series (retinography) Supplementary file8 (TIF 859 KB) [file 10792_2023_2656_MOESM8_ESM.tif]
