# Supplementary material for: First-line management of necrotizing herpetic retinitis by prioritizing the investigation of immune status and prognostic factors for poor visual outcomes
Source: Int Ophthalmol. 2023 Mar 15;43(7):2545–56. doi: 10.1007/s10792-023-02656-8 (PMC10313533; doi:10.1007/s10792-023-02656-8)
Supplement: Supplementary file 5 — Supplementary file5 (DOCX 46 KB) [file 10792_2023_2656_MOESM5_ESM.docx]

**Supplementary Fig 4** Summary of the different viral infections identified by anterior chamber paracentesis according to diagnosis of acute retinal necrosis and progressive outer retinal necrosis.
